# Tank-based bacterial profiling identifies basin-wide white band disease pathogen candidate and no bacterial associations with coral disease resistance

**DOI:** 10.1093/ismeco/ycaf247

**Published:** 2025-12-19

**Authors:** Emily C Trytten, Brecia A Despard, Jason D Selwyn, Steven V Vollmer

**Affiliations:** Department of Marine and Environmental Sciences, Northeastern University, Nahant, MA, United States; Department of Marine and Environmental Sciences, Northeastern University, Nahant, MA, United States; Department of Marine and Environmental Sciences, Northeastern University, Nahant, MA, United States; Genomics Core Laboratory, Texas A&M University-Corpus Christi, Corpus Christi, TX, United States; Department of Marine and Environmental Sciences, Northeastern University, Nahant, MA, United States; Department of Biological Sciences, Florida Atlantic University, Boca Raton, FL, United States; School of Environmental, Coastal, and Ocean Sustainability, Florida Atlantic University, Boca Raton, FL, United States

**Keywords:** white band disease, staghorn coral, Acropora cervicornis, Cysteiniphilum litorale, disease resistance

## Abstract

White band disease (WBD) has decimated the Caribbean staghorn coral, *Acropora cervicornis*, since its emergence in 1979, but its etiology remains unknown. Numerous WBD pathogen candidates from over nine bacterial families have been implicated, with a multi-year field study recently identifying *Cysteiniphilum litorale* as the likely pathogen. Here, we use 16S rRNA gene amplicon sequencing to profile changes in the bacterial communities in a tank-based transmission experiment in the Florida Keys using 50 nursery-raised staghorn coral genotypes with varying disease resistances to determine whether any bacteria in the native staghorn coral microbiomes were associated with WBD resistance and to identify bacterial amplicon sequencing variants (ASVs) associated with WBD exposure and transmission. We found no significant associations, positive or negative, between any bacterial ASV, genus, or family and disease resistance in native staghorn coral microbiomes but did identify nine bacterial ASVs strongly associated with disease outcome in the tank-based transmission experiment. ASV 65, classified as *Cysteiniphilum litorale*, showed strong disease associations consistent with pathogenicity, including being significantly associated with WBD transmission within disease-exposed tanks (i.e. more abundant on diseased fragments) and being significantly more abundant on the diseased experimental dose than the healthy dose. The V3-V4 16S rRNA gene sequence for ASV 65 differed by only 1 of 415 bp from the *C. litorale* ASV identified as the putative WBD pathogen in the recent multi-year study from Panama, suggesting a rare Caribbean-wide strain-level pathogen association. Eight additional disease-associated ASVs were identified as potential opportunistic pathogens and included ASVs from the families *Vibrionaceae* and *Colwelliaceae*.

## Introduction

The rate and severity of marine epizootics has increased dramatically in the last few decades [1, 2], partially in response to rapidly warming ocean temperatures [3, 4]. Emergent coral diseases have caused unprecedented mortality on Caribbean coral reefs with stony coral tissue loss disease currently impacting more than 20 Scleractinian coral species [5–7] and white band disease (WBD) decimating populations of Caribbean acroporid corals since 1979 [8, 9]. Despite the devastating impacts of coral diseases on tropical reefs [10–12], the etiology of most coral diseases remains unknown, with coral pathogens conclusively identified for only six of the 20-plus known coral diseases [13, 14]. High coral microbial diversity [15, 16], the complexity of the coral holobiont [17], and the high frequency of opportunistic pathogens in marine systems [18, 19] have made primary pathogen identification and cultivation difficult [20, 21] and culture-independent genetic analyses often identify tens to hundreds of disease-enriched bacteria [22, 23] or candidate bacterial pathogens [24]. This lack of clear putative coral pathogens complicates efforts to manage and treat coral disease outbreaks.

A major difficulty in identifying putative pathogens for marine diseases is distinguishing between primary pathogens and opportunistic or secondary pathogens, especially given the high prevalence of opportunistic pathogens in marine systems [18, 19]. Primary pathogens are disease agents capable of causing disease regardless of the host’s health state or environmental conditions (e.g. *Pseudoalteromonas agarivorans* in the sponge *Rhopaloeides odorabile* [25, 26]). In this paper, we refer to bacteria that are believed to be primary pathogens but for whom causality has not yet been experimentally proven as putative pathogens and bacteria who are associated with the diseased state but who are unlikely to be pathogens as unlikely pathogens. Secondary pathogens cause disease in weakened hosts who have already been infected by a primary pathogen, resulting in a coinfection (e.g. sea lice and *Piscirickettsia salmonis* in Atlantic salmon [27]). Opportunistic pathogens, on the other hand, are able to cause disease in any host, healthy or diseased, when certain favorable conditions are met. These favorable conditions can include environmental factors (e.g. heat-stress [28] or eutrophication [29]) or a weakened host immune system resulting from an ongoing infection, in which case an opportunistic pathogen is also acting as a secondary pathogen. When conditions are not favorable for pathogenicity, they act as harmless commensal members of the microbiome [30]. Opportunistic pathogens can also be described as intrinsic pathogens, as the disease-causing agent originated from within the native healthy microbiome, whereas primary pathogens can be termed extrinsic, as the disease-causing agent originated from outside the native healthy microbiome. Primary and secondary/opportunistic pathogens can exhibit similar behaviors in active disease lesions (e.g. high abundance, expression of virulence factors), which greatly complicates differentiating them.

A new term for describing disease systems has recently emerged in response to deepening understandings of disease dynamics and the limitations of the traditional one pathogen-one disease paradigm [31, 32]: a pathobiome is the whole microbial community associated with a diseased host that has a direct role in causing disease [21, 31]. Pathobiomes include interactions between primary, secondary, and opportunistic pathogens, better representing how dysbiotic bacterial communities influence disease dynamics and differ from healthy microbiomes [21, 32, 33]. Examining disease-associated microbial communities through the pathobiome lens therefore accounts for disease systems with multiple co-infecting pathogens and systems where the secondary and/or opportunistic pathogens modulate the activity of the primary pathogen [31]. The pathobiome concept of disease is highly applicable to coral diseases, especially given the known frequent associations between opportunistic *Vibrio* spp. and diseased microbiomes [34]. Examining the pathobiome will deepen our understanding of the role of such opportunists in pathogenesis.

A prime example of a coral disease outbreak is WBD, which infects the two Caribbean acroporid species, *Acropora cervicornis* [35] and *A. palmata* [9], as well as their hybrid [36, 37]. The WBD epizootic has decimated up to 95% of Caribbean acroporid populations [8, 9], rendering them critically endangered [38]. WBD is caused by a bacterial infection whose transmission can be arrested with the addition of broad-spectrum antibiotics [39, 40] and by inhibiting bacterial quorum sensing [41]. Two forms of WBD have been described based on differences in disease lesion appearances—healthy tissue directly borders bare skeleton in type I [9] whereas a region of bleached tissue separates them in type II [35]—even though both types of disease signs are often seen together on a single infected colony [42, Vollmer, pers. obs.]. Many authors use the term WBD to describe this infection [8, 39, 43–46], as we do here, while others favor the more general term of rapid tissue loss [42, 47].

Although the Henle-Koch postulates have not been fulfilled for WBD, early disease associations showed a strong relationship between WBD infection and the genus *Vibrio*. *Vibrio charcharia* (now *Vibrio harveyi*) was initially proposed as the putative pathogen [35] and *in situ* exposure of an uncharacterized *Vibrio* sp. onto healthy corals elicited WBD-like disease signs in *A. cervicornis* [48]. More recent 16S rRNA gene amplicon sequencing studies have implicated a wide range of disease-associated bacterial taxa across more than nine bacterial families. Our previous transmission experiments and field-based surveys of *A. cervicornis* identified amplicon sequencing variants (ASVs)/operational taxonomic units (OTUs) from the families *Flavobacteriaceae*, *Vibrionaceae* [24, 41], *Campylobacteraceae*, *Francisellaceae,* and *Pasteurellaceae* [43] as pathogen candidates for WBD. In situ grafting experiments on *A. cervicornis* and *A. palmata* in Florida identified ASVs from the families *Sphingomonadaceae*, *Cryomorphaceae*, *Rhodobacteraceae*, and *Vibrionaceae* as being disease-associated, with the ASV from *Sphingomonadaceae*, identified as *Sphingobium yanoikuyae*, proposed as a potential putative pathogen [49]. Most recently, Selwyn *et al.* [44] compared 16S rRNA gene amplicon sequencing data from 269 healthy and 143 diseased field-collected *A. cervicornis* samples across multiple years using a novel ensemble machine learning approach and identified two ASVs– a *Cysteiniphilum litorale* and a *Vibrio* sp. – as likely pathogens for WBD. They confirmed these results with a more traditional tank transmission experiment and differential abundance analysis approach, which identified the same two ASVs as pathogen candidates. Selwyn *et al.* [45] further examined how antibiotic pretreatment prior to disease exposure impacts bacterial community composition and disease outcomes. They found that antibiotic pretreatment on healthy staghorn corals significantly reduced the abundance of the intrinsic WBD-associated *Vibrio* ASV, thereby suppressing WBD transmission in disease-exposed corals and suggesting that this *Vibrio* sp. is an opportunistic pathogen. The putative extrinsic pathogen *C. litorale* was not detected on healthy corals prior to WBD-exposure and abundances were, therefore, unaffected by the antibiotic treatment.

*A. cervicornis* genotypes also display phenotypic variation in disease resistance to WBD [50–52] with more than 20% of individuals being highly resistant to infection [52]. Using a genome-wide association study on *A. cervicornis* from tank-based transmission experiments in Florida and Panama, Vollmer *et al.* [52] demonstrated that 6.1% of variation in WBD resistance is genetic and developed a polygenic model that can accurately predict disease resistance from as few as 10 key genetic loci. The presence of probiotic microbes in the coral holobiont has also been suggested as a possible mechanism for disease resistance, with Myxococcales [49] and *Endozoicomonas* [43] being previously identified as potential probiotics in *A. cervicornis*. Conversely, the intracellular bacterial parasite *“Candidatus* Aquarickettsia rohweri*”* has been linked to increased disease susceptibility in Florida [53].

In this study, we obtained 16S rRNA gene amplicon sequencing data from the 50 nursery-raised staghorn coral genotypes from Florida used in Vollmer *et al.*’s [52] tank-based transmission experiment in order to determine whether any bacterial ASVs initially present on these staghorn coral genotypes were positively or negatively correlated with their observed disease resistance and identify bacterial ASVs that were associated with disease exposure and outcome. Positive and negative bacterial associations with disease resistance were tested with correlation tests for 16 staghorn coral genotypes displaying a range of variation in disease resistance. We applied linear mixed-effects regressions to longitudinal samples of 46 *A. cervicornis* genotypes to identify bacterial ASVs that differed significantly due to time, disease exposure, and/or disease outcome. Using *post hoc* contrasts, we differentiated putative primary pathogens from general disease-associated opportunists based on their differential abundances, resulting in a reduced list of pathogen candidates.

## Materials and methods

### Tank-based transmission experiment

**Figure 1:**
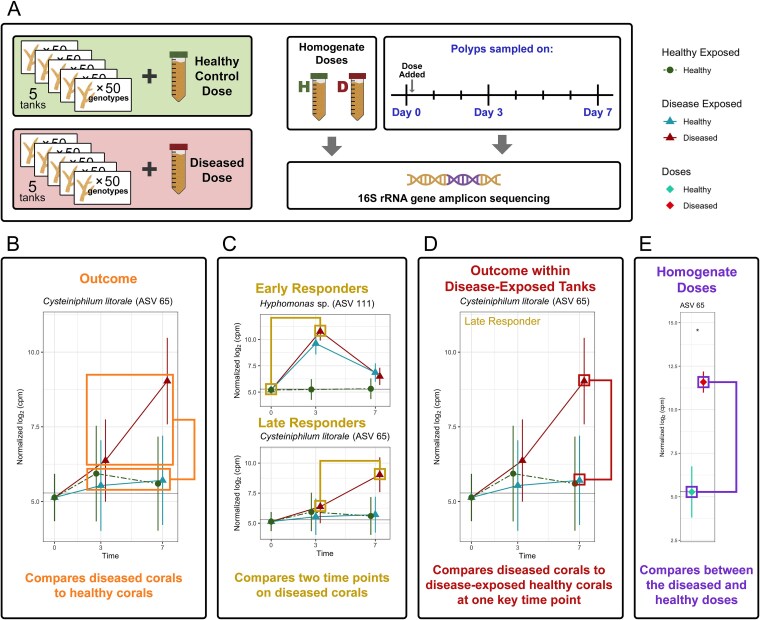
Schematic showing (A) the experimental design of the tank transmission experiment and (B–E) representative examples of the differential abundance comparisons used to identify putative pathogens and opportunists. (A) Five hundred coral fragments from 50 genotypes were divided evenly between 10 tanks, with half of the tanks being exposed to a healthy tissue homogenate and the other half exposed to a diseased tissue homogenate. Samples were taken for 16S rRNA gene amplicon sequencing from the tissue homogenates as well as day 0 (nursery-collected fragments) and days 3 and 7 post-exposure to the homogenate doses. Differential abundance comparisons include differences based on (B) disease outcome, (C) early vs. late growth response on corals that contract disease, (D) disease outcome within disease-exposed tanks, and (E) diseased vs. healthy homogenate doses.

A tank-based transmission experiment was performed at the Florida Keys Marine Laboratory in June 2021 using 550 fragments from 50 genotypes of *A. cervicornis* collected from the Coral Restoration Foundation nursery in Tavernier, Florida, to calculate genotype-level disease resistance scores (details published in [52]) and examine changes over time in the coral microbial communities as a result of disease exposure and infection (Fig. 1A). Coral Restoration Foundation’s *in situ* nursery consists of PVC pipe “trees,” each containing fragments of one genotype, where coral fragments are grown suspended in the water column using fishing line. One sample of each genotype (day 0) was taken immediately after collecting fragments from the nursery to examine the unaltered native microbiomes. Microbial diversity differs significantly between coral genotypes [54, 55], so sampling was performed across genotypes (i.e. genotype-level replication) to capture most of the microbial diversity present in the nursery and establish a baseline average microbiome for healthy nursery-raised *A. cervicornis*. One limitation is that this assumes that the average microbiome composition of these day 0 samples is representative of healthy corals in the nursery environment, so it may underestimate the true microbial diversity within a genotype. The remaining 10 independent replicate fragments per genotype were distributed across 10 18-liter recirculating tanks held at ambient temperatures such that each tank contained one fragment from all 50 coral genotypes (50 fragments per tank total). Each tank was dosed with 50 ml of a healthy or diseased tissue slurry (hereafter referred to as healthy and diseased “doses”), with five replicate tanks being “healthy-exposed” and five replicate tanks being “disease-exposed.” These homogenate doses were created by using a WaterPik containing filtered seawater to remove the tissue from five healthy and five diseased nursery-collected fragments of *A. cervicornis*, then normalizing the optical density across the two slurry types. Coral fragments in the tanks were lesioned with a WaterPik to facilitate transmission. Three polyps were sampled from each fragment on day 3 after exposure and again on either day 7 or when the fragment developed disease signs, whichever occurred first. Polyps were sampled from the disease interface for diseased individuals. Fragments were monitored for disease signs at least twice per day and fragments displaying symptoms of disease were removed from the tanks to prevent amplification of transmission. Sampled polyps were stored in the preservative DNA/RNA Shield (Zymo Research, Irvine, CA, USA) at −20°C for less than two months before performing DNA extractions.

### 16S rRNA gene amplicon sequencing

Genomic DNA was extracted from each coral sample using a GenElute Bacterial Genomic DNA kit (Sigma-Aldrich, Burlington, MA, USA). The V3-V4 region of the 16S rRNA gene was amplified using the following primer pairs: (i) S-D-Bact-0341b-S-17 [5′-CCTACGGGNGGCWGCAG-3′]; and (ii) S-D-Bact-0785-a-A-21 [5′-GACTACHVGGGTATCTAATCC-3′] [56, 57]. Each 23 μL PCR reaction was performed using 1 μL DNA, 1.25 μL each 10 mM primer, 12.5 μL 2X Phusion Mix (Thermo Fisher Scientific, Waltham, MA, USA), and 7 μL molecular biology grade water. The PCR program consisted of initial denaturation for 1 min at 98°C, 28 cycles of denaturation (30 s at 98°C), annealing (30 s at 63°C), and extension (30 s at 72°C), and a final extension phase of 5 min at 72°C. PCR products were then cleaned using a ZR-96 DNA Clean-Up kit (Zymo Research, Irvine, CA, USA). Indexes for sequencing were added during a separate PCR reaction using 5 μL purified PCR product, 2.5 μL each index primer, 12.5 μL 2X Phusion Mix (Thermo Fisher Scientific, Waltham, MA, USA), and 2.5 μL molecular biology grade water. The PCR program consisted of initial denaturation for 1 min at 98°C, 12 cycles of denaturation (30 s at 98°C), annealing (30 s at 55°C), and extension (30 s at 72°C), and a final extension phase of 5 min at 72°C. Indexed PCR products were cleaned and normalized using a SequalPrep Normalization Plate kit (Thermo Fisher Scientific, Waltham, MA, USA), then pooled and concentrated using a DNA Clean & Concentrator kit (Zymo Research, Irvine, CA, USA). These amplified products were then sequenced on two runs of Illumina MiSeq 2x300 bp sequencing. The 16S rRNA gene reads were quality trimmed, overlapped, and assembled using the dada2 denoising algorithm and pipeline in R v4.4.1 [58, 59] to generate ASVs, then chimeras were removed. This pipeline consisted of the following functions and nondefault parameters: filtering and trimming with filterAndTrim() using trimLeft = 25, truncLen = c(250, 230), maxEE = c(2, 2); dereplication with derepFastq(); error rates were estimated with dada() on the first 40 samples using err = NULL, selfConsist = TRUE; sample inference was run with dada() using the estimated error rates and pool = TRUE; paired reads were merged with mergePairs(); an ASV table was generated with makeSequenceTable(); and chimeras were removed with removeBimeraDenovo(). ASV taxonomy was assigned first using blca, a Bayesian taxonomic classifier based on the NCBI microbial 16S rRNA gene full-length database (accessed 02/05/2024), where ASVs were classified to the lowest taxonomic rank with at least 80% classification confidence [60]. Any ASVs that did not have confident classifications below the class level were then reclassified using the SILVA SSU database r138 [61] using a threshold of 50. decipher [62] was then used to align ASV sequences and phangorn [63] was used to generate a neighbor-joining tree of these aligned sequences. A phyloseq object [64] was then created using the ASV table, taxa table, and 16S rRNA gene tree, which was merged with the sample metadata and used for downstream analyses.

Data was filtered to retain only ASVs within the domain Bacteria and to remove chloroplast, mitochondrial, and cyanobacterial sequences as potential host/symbiont contamination [65, 66]. This removed 52 Cyanobacteria ASVs, all of which would have been filtered out downstream regardless as a result of low prevalence. Samples were then filtered to contain a minimum of 1000 16S rRNA gene reads per sample and to only include coral genotypes that had longitudinal data across days 3 and 7. The day 0 samples consisted of a subset of 16 staghorn coral genotypes chosen to represent a wide range of disease resistance values. After calculating alpha diversity metrics, ASVs in fewer than 20% of samples were removed due to low prevalence and ASVs that were not present in either homogenate dose or on the day 0 samples were removed as potential contaminants originating from the tank environment. This 20% prevalence cut-off was chosen because 27.3% of the samples sequenced were from diseased corals and we would expect a true pathogen to be present in all diseased individuals according to the Henle-Koch disease postulates. This filtering step removed the majority of ASVs that were not consistent across all diseased samples and, therefore, could not be potential pathogens. Choosing data filters is a balance between removing noise that will interfere with properly detecting patterns and not filtering so stringently that potentially important ASVs are removed prior to analysis and not detected. Although the filters applied here were chosen thoughtfully, the possibility exists that biologically relevant ASVs are missing from the final filtered dataset used in the differential abundance analysis. This filtered dataset was used in all downstream analyses except community composition visualization and alpha diversity analyses, which both used the unfiltered dataset. Read counts were normalized using the trimmed mean of M-values with singleton pairing method in edgeR, which also considers effective library size, to account for differing levels of sequencing depth, a normalization process termed ELib-TMM [67, 68]. These normalized counts plus a pseudo-count of 0.5 were then transformed into log_2_ counts per million reads, which were used in all downstream analyses. This normalization method shows similar performance to the frequently used ANCOM-BC normalization method while also allowing for full contrasts and *post hoc* tests within modeling frameworks [69], such as the linear mixed-effects models with interactions and *post hoc* analyses employed here. In general, the random effect of tank in the following models was applied only to the day 3 and day 7 samples, all of which were incubated in our experimental tank setup. Day zero samples were nursery-collected and thus the microbiome composition of each fragment is relatively independent compared to samples sharing the same aquarium unit. All false discovery rate (FDR) corrections were made using the Benjamini & Hochberg method [70] and a *P*-value threshold of 0.05.

### Community level analyses

Overall trends in microbial community composition between timepoints, exposure types, and disease outcomes were visualized using the R package Fantaxtic [71]. The complete unfiltered dataset was used for this visualization to fully capture the observed microbiome diversity. The 11 most abundant bacterial orders across all samples and the four most abundant genera within each order were visualized, with all other bacterial orders grouped into the “Other” category.

Two alpha diversity metrics—the Shannon diversity index and Simpson’s dominance index—were calculated using the microbiome package in R [72] and analyzed via a linear mixed-effects regression to examine differences in alpha diversity related to sampling time, exposure, and disease outcome, with genotype and tank as random effects. Abundance count data was first rarefied to the lowest sequencing depth of any sample using seed number 68748 to account for uneven library sizes between samples.

Differences between samples driven by time, exposure, and disease outcome were visualized via a non-metric multidimensional scaling (NMDS) plot based on microbial distances between the sampled fragments, which was calculated using the Bray–Curtis dissimilarity index [73]. Differences in microbiomes across these three factors, as well as across genotype and tank, were examined using a permutational ANOVA with 10 000 permutations and seed number 68748. These beta diversity analyses were performed on the set of filtered ASVs.

### Bacterial associations with disease resistance

To examine whether the bacterial consortium in a native *A. cervicornis* microbiome can predict an individual’s observed level of disease resistance, a Kendall’s correlation test between normalized 16S rRNA gene abundances and disease resistance scores was run for nursery-collected day 0 fragments. Both positive associations, such as beneficial microbes, and negative associations, like parasites or lurking opportunists, were considered. These correlation tests were run at the ASV, genus and family levels to detect associations across multiple taxonomic ranks, and *P*-values were FDR-corrected. Observed disease resistance was calculated using a Cox proportional hazards model and published in Vollmer *et al.* [52]. Taxonomic groups with previously documented associations to WBD resistance were also specifically examined.

### Identifying key amplicon sequencing variant associations

To identify pathogens, opportunists, and potentially beneficial microbes, we ran weighted linear mixed-effects regression models for each ASV with fixed effects of sampling time, exposure, and disease outcome and random effects of genotype and tank. Observation-level weights were calculated using voomWithDreamWeights in the variancePartition package [74–76]. Only ASVs showing a significant main effect of treatment type (sampling time, exposure, and/or disease outcome) were retained for further analysis. ASV relative abundances in the healthy versus diseased homogenate doses were examined using linear models. All *P*-values for the main effects in both models were FDR-corrected. We then set up two-sided *post hoc* contrasts for transplant effect, exposure, and disease outcome to investigate key patterns in ASV relative abundances (Supplementary Table S1).

First, we used a transplant effect contrast to filter out ASVs whose abundances strongly shifted on healthy-exposed corals as a result of being transplanted into the tank environment. We compared ASV relative abundances on the nursery-collected day 0 samples to the average of the tank-incubated healthy control samples on days 3 and 7. This identified ASVs whose abundances changed significantly between healthy fragments in the *in situ* nursery and healthy control fragments in the tank environments, where significant differences between the two can be largely attributed to their transplant into the tank environment and bacterial strains responding either well or poorly to this new environment. A significant positive association for transplant effect identifies ASVs that thrive in the tank environment whereas a negative association identifies ASVs with poor growth and survival in the tanks. These transplant effect-associated ASVs were removed from downstream analyses to minimize false positives.

Next, we identified putative opportunists via the disease exposure contrast by comparing healthy-exposed and diseased-exposed fragments across days 3 and 7, regardless of final disease outcome. The directionality of this contrast indicated whether the significant ASVs were associated with exposure to the healthy or the diseased dose. ASVs that are significant for exposure to the diseased dose represent opportunists, as they are abundant on both diseased and healthy corals that were exposed to the diseased homogenate. Conversely, ASVs associated with the healthy exposure (i.e. dose) likely represent commensalists that are outcompeted or killed by opportunists in a compromised host environment.

We then used a disease outcome contrast to compare samples that contracted disease to all fragments that remained healthy with directionality indicating whether the ASV is associated with a healthy or diseased outcome (Fig. 1B). ASVs that show a significant positive association with a diseased outcome represent putative pathogen candidates whereas ASVs significantly positively associated with the healthy outcome are likely core members of a healthy microbiome that are lost during infection or potentially beneficial microbes that may confer resistance to infection.

To further eliminate latent opportunists from our primary pathogen candidates, additional criteria were applied to the ASVs showing a significant association with a diseased outcome. First, ASVs were categorized into two groups based on when their abundance spiked on diseased corals relative to the emergence of visible disease lesions: early and late responders (Fig. 1C). Early responders significantly increased in abundance on diseased corals between days 0 and 3, prior to the onset of disease symptoms. Late responders showed this significant increase between days 3 and 7, during the same period of time in which disease signs manifested. Within disease-exposed fragments, abundances were then compared between individuals that contract disease versus those that remain healthy at the timepoint of the highest bacterial growth; for early responders, this comparison was made on day 3 whereas day 7 was examined for late responders (Fig. 1D). Requiring bacterial abundances to be significantly higher on diseased fragments than disease-exposed healthy fragments at this key timepoint removed latent opportunistic bacteria that were not identified by the exposure contrast because they show a signature of opportunism at only one key timepoint rather than throughout the experiment. This limited our candidate pool to only include strong pathogen candidates and WBD-associated opportunists that display similar growth signatures as a putative pathogen.

Finally, we expected that a pathogen would be highly abundant in the diseased homogenate dose, given that the dose was created from tissue with active WBD lesions. Putative pathogens were therefore required to have elevated abundances on the diseased dose compared to the healthy dose (Fig. 1E). This criterion separates putative pathogens from WBD-associated opportunists and provides a substantially reduced list of candidate pathogens.

## Results

**Figure 2:**
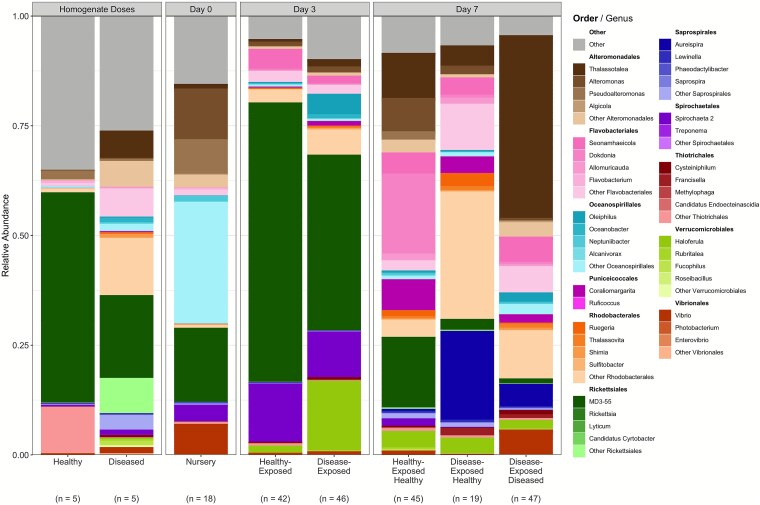
The relative abundances of the 11 most abundant microbial orders across all samples, indicated by color, with shades of each color indicating the four most abundant genera within each order. The complete, unfiltered dataset was used to generate these relative abundances. Samples were grouped based on timepoint and the exposure type and/or disease outcome. Sample sizes for each bar are listed below the x-axis labels.

A representative subset of 234 samples were chosen for 16S V3-V4 rRNA gene amplicon sequencing, comprised of 206 samples total across 48 genotypes from days 3 to 7 of the tank-based experiment, 18 nursery-collected day 0 samples across 18 genotypes, and 10 samples of the homogenate doses (five healthy dose and five diseased dose samples). All 16S rRNA gene data was filtered for a minimum of 1000 reads per sample (Supplementary Fig. S1) and for genotypes that showed full temporal presence in the tank environment (i.e. present post-filtering on day 3 and day 7 in any tank). This resulted in 195 samples across 46 genotypes from the tank transmission experiment and 16 nursery-collected day 0 samples from 16 genotypes that span a diversity of disease resistance scores. 10 of the 250 disease-exposed fragments (4.0%) showed visible disease signs on day 3, whereas 133 of the 250 disease-exposed fragments (53.2%) displayed visible WBD signs by day 7. The 16S rRNA gene dataset consisted of 5716 bacterial ASVs from 49 classes, 102 orders, and 219 families and 411 genera (Fig. 2; Supplementary Data S1). This was reduced to 254 ASVs after filtering out ASVs found in less than 20% of samples and ASVs that did not originate on either of the homogenate doses (representing potential transmissible pathogens) nor in the native day 0 microbiome (as an intrinsic member of the coral holobiont). Homogenate doses contained 199 of the 254 ASVs and spanned 14 classes, 29 orders, 48 families, and 61 genera.

### Community level analyses

Large variations in microbial relative abundances were observed between timepoints, exposure types, and disease outcomes (Fig. 2). The 11 most abundant bacterial orders constituted a large proportion of the observed diversity in the tank environment, ranging from 90.1% (day 3, disease-exposed) to 95.6% (day 7, disease-exposed diseased).

**Figure 3:**
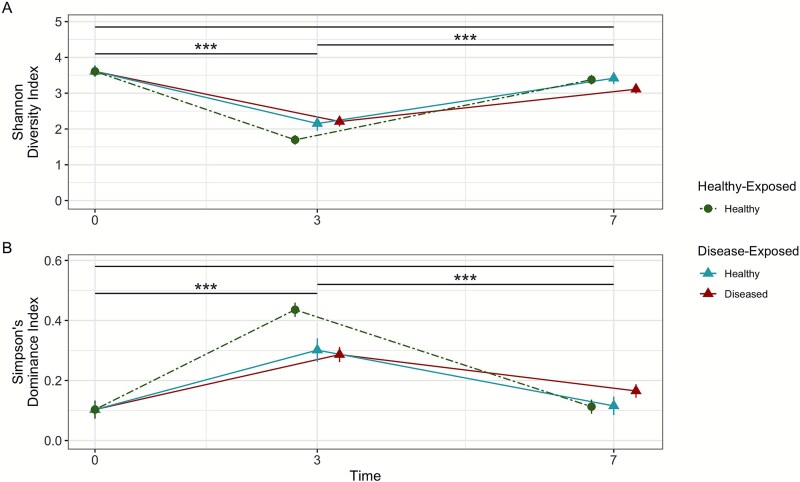
Rarefied alpha diversity metrics examining (A) Shannon diversity and (B) Simpson’s dominance. Shapes and colors represent the exposures and health states of each group. Stars over horizontal black lines indicate significance between the timepoints (*P <* .05: ^*^; *P <* .01: ^**^; *P <* .001: ^***^). Error bars represent standard error.

Both alpha diversity metrics rarefied to the lowest sequencing depth of any sample (1029 reads) – the Shannon diversity index and Simpson’s dominance index – varied significantly by sampling time (Shannon: t_27.5_ = −5.73, *P* < .001; Simpson: t_42.5_ = 4.78, *P <* .001) and not by disease exposure or disease outcome (Supplementary Table S2). Shannon diversity dropped significantly from 3.61 ± 0.15 SE for day 0 to 2.02 ± 0.18 SE on day 3 of the tank experiment (t_79.2_ = −8.88, *P <* .001) and then increased significantly to 3.30 ± 0.10 SE on day 7 (t_164_ = 13.4, *P <* .001; Fig. 3A). Simpson’s dominance index increased significantly from 0.10 ± 0.03 SE for day 0 nursery samples to 0.34 ± 0.04 SE on day 3 (t_106_ = 6.74, *P <* .001) and then decreased significantly to 0.13 ± 0.02 on day 7 (t_164_ = −10.7, *P <* .001; Fig. 3B). The increase in Shannon diversity and corresponding drop in Simpson’s dominance were associated with a spike in *MD3–55* (newly termed “*Candidatus* Aquarickettsia”) relative abundance at day 3 of the tank experiment (Fig. 2). These alpha diversity metrics trended back towards their initial values with the decrease in relative abundance of MD3–55 by day 7.

**Figure 4:**
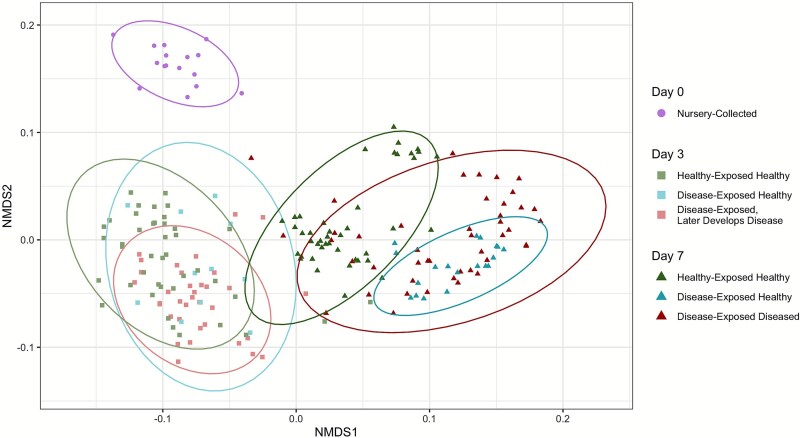
Community composition via NMDS considering tank, time, exposure, disease outcome, and genotype. Ellipses represent 95% confidence intervals. Sampling day is represented by shape whereas colors represent exposure type and disease outcome.

NMDS and permutational ANOVA analyses show that the composition of the microbial communities differed significantly across all factors (Fig. 4, Supplementary Table S3). Time accounted for 36.03% of the observed variation (F_(2, 153)_ = 90.10, *P <* .001), genotype accounted for 13.55% (F_(45,153)_ = 1.51, *P <* .001), experimental tank for 6.29% (F_(6, 153)_ = 5.24, *P <* . 001), disease outcome for 5.80% (F_(1, 153)_ = 28.99, *P <* . 001), and exposure for 3.46% (F_(1, 153)_ = 17.30, *P <* .001).

### Bacterial associations with disease resistance

We used Kendall’s correlation tests to determine whether any bacterial ASV, genus or family initially present on the 16 staghorn coral genotypes sampled on day 0 could predict their observed disease resistance scores, which ranged from 0.03 to 0.64 (average: 0.41 ± 0.05 SE) where higher values correspond to greater resistance to WBD infection. No significant positive or negative associations were detected between observed disease resistance and any bacterial ASV, genus or family, including in the order Myxococcales (r = 0.07, *P* = .96) and the genus *Endozoicomonas* (not present at detectable levels), which have previously been positively associated with healthy corals, or in the genus *MD3–55* (sensu “Ca. Aquarickettsia”; r = 0.02, *P =* .97), which has previously been positively associated with increased susceptibility to WBD.

### Differentially abundant amplicon sequencing variants by disease exposure, outcome, and transplant effect

Our weighted linear mixed-effects model analyses identified 118 ASVs that differed significantly in their relative abundances across one or more main effects (transplant effect, disease exposure, or disease outcome; Supplementary Fig. S2). Ninety-eight ASVs differed due to transplant effects and were excluded from downstream analyses (Supplementary Fig. S2). No ASVs differed by exposure type (healthy vs. diseased) in the tank environment, whereas 20 ASVs differed significantly by disease outcome. Of these, 18 ASVs were more abundant on WBD-infected fragments and two ASVs—one each from the genera *Thalassoglobus* (ASV 301) and *Arenicella* (ASV 127)—were associated with healthy corals (Supplementary Fig. S2).

**Figure 5:**
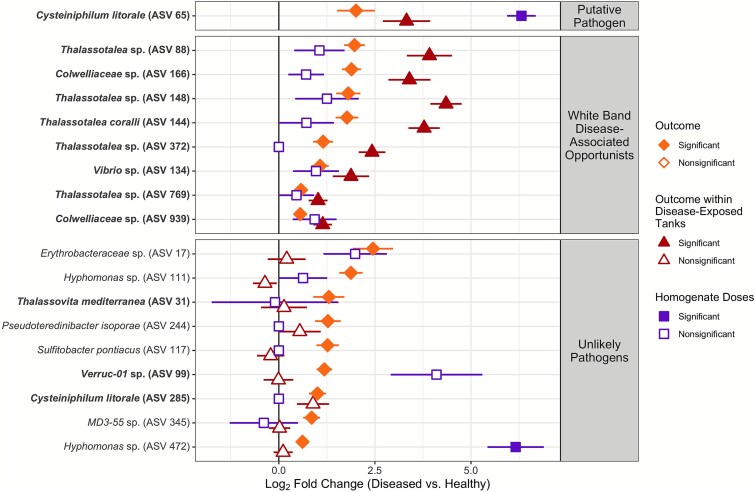
The log_2_ fold change in abundance between diseased and healthy treatments for the 18 disease-associated ASVs showing a significant effect of outcome, including the top pathogen candidate, *Cysteiniphilum litorale* (ASV 65). Outcome (orange diamonds) indicates the difference between fragments that contract disease and those that remain healthy. Outcome within disease-exposed tanks (red triangles) compares between infected fragments and disease-exposed fragments that remained healthy. The homogenate doses contrast (purple squares) compares the homogenate doses added to the tanks on day 0. Filled shapes indicate a significant difference. Bolded ASVs are late responders whereas nonbolded ASVs are early responders. Error bars represent standard error.

For the 18 disease-associated ASVs, we applied additional post hoc tests to further refine the pathogen candidate list (Supplementary Table S4). We first classified these disease-associated ASVs into early or late responders by testing whether their abundances increased significantly over time on diseased corals, with early responders increasing in abundance between days 0 and 3 and late responders increasing between days 3 and 7. Six ASVs were categorized as early responders, 12 ASVs were late responders, and no ASVs responded continuously. We then assessed whether their abundances differed between healthy and diseased corals within disease-exposed tanks on day 3 (early responders) or day 7 (late responders). None of the six early responders differed significantly by disease outcome in the disease-exposed tanks at day 3, but nine of the 12 late responders differed significantly due to disease outcome at day 7 and thus represent the most likely pathogen candidates (Fig. 5, red triangles).

The nine late responding pathogen candidates include one ASV from the genus *Cysteiniphilum* (ASV 65), seven ASVs from the family *Colwelliaceae* – five of which are in the genus *Thalassotalea* (ASV 88, 144, 148, 372, 769) and two of which are unclassified at the genus level (ASV 166 and ASV 939) – and one ASV from the genus *Vibrio* (ASV 134; Supplementary Fig. S3). Only ASV 65, classified as *Cysteiniphilum litorale*, also differed significantly between the healthy and diseased experimental doses used in the transmission experiment (Fig. 5, purple squares).

## Discussion

### Initial staghorn coral microbiomes do not predict observed disease resistance

Beneficial coral microbes are thought to play a key role in promoting holobiont health and resisting infection [77]. Ritchie [78] identified numerous antibiotic-producing resident bacteria in *A. palmata*, the sister species to *A. cervicornis*, that inhibit the growth of pathogenic bacteria. Ushijima *et al.* [79] recently isolated the probiotic *Pseudoalteromonas* sp. strain McH1–7, which fully arrests the transmission of stony coral tissue loss disease on *Montastraea cavernosa*. In *A. cervicornis*, the order Myxococcales [49] and the genus *Endozoicomonas* [43] have been positively associated with healthy corals in disease transmission assays, whereas parasitic *“Candidatus* Aquarickettsia rohweri” have previously been found at high abundances on WBD susceptible *A. cervicornis* in Florida [53]. Yet, we failed to detect any significant positive or negative associations between host disease resistance and any bacterial ASV, genus or family in the native microbiomes of the 16 staghorn coral genotypes sampled at day 0, including no significant positive associations with the taxa Myxococcales and *Endozoicomonas* or significant negative associations with parasitic “Ca. Aquarickettsia.” Instead, “Ca. Aquarickettsia” (*MD3–55*) ASVs spiked in abundance on day 3 regardless of disease exposure and then dropped off towards day 7, which is consistent with *MD3–55* sp. being a highly infectious parasite [80]. Interestingly, “Ca. Aquarickettsia” dominate nursery-raised *A. cervicornis* microbiomes from Florida (this study, [49, 81]) and the Cayman Islands [54, 82], but are at very low prevalence in wild staghorn populations from Panama [44]. This may reflect broader phylogeographic differences in staghorn coral microbiomes and/or different infection histories of this bacterial parasite, including between nursery-raised and wild populations.

### Putative white band disease pathogens and opportunists

Of the nine late responding pathogen candidates identified here, only ASV 65—classified as *Cysteiniphilum litorale* in the family *Fastidiosibacteraceae*—also differed significantly in abundance between the diseased and healthy homogenate doses used in the transmission experiment. This suggests that this ASV is an extrinsic pathogen that was introduced into the tanks at higher abundances on the diseased dose. *Cysteiniphilum litorale* was first isolated from seawater off the coast of southern China [83] and the closely related *Cysteiniphilum* sp. QT6929 (97% similarity to ASV 65; 401/415 bp) was cultured from a human skin infection resulting from an injury while handling estuarine shrimp [84, 85], demonstrating the pathogenic potential of this genus. The family *Fastidiosibacteraceae* is closely related to the highly pathogenic family *Francisellaceae* [86, 87] and published *Cysteiniphilum* genomes contain multiple virulence factors, including a portion of the Francisella pathogenicity island [84]. At the close of the tank transmission experiment, ASV 65 was detected on 71.35% ± 0.14% SE of diseased fragments of *A. cervicornis* compared to only 11.26% ± 0.10% SE of all healthy fragments, regardless of exposure. Similarly, the prevalence of this ASV on the putatively healthy nursery-collected samples was only 6.10% ± 0.08% SE.

*Cysteiniphilum litorale* has previously been associated with WBD [43–45, 88], including several associations prior to the separation of *Fastidiosibacteraceae* from the family *Francisellaceae* [87]. Most significantly, Selwyn *et al.* [44] identified *C. litorale* as the top WBD pathogen candidate in their machine learning analyses of two years of field sampling in Panama and an independent tank-based transmission experiment, which supported its link to disease transmission. Their *C. litorale* ASV25 differed from our ASV 65 by only one out of 415 nucleotides (0.24%). Therefore, this implicates for the first time two highly similar strains of *C. litorale* across the Caribbean as the most likely WBD pathogen. *Cysteiniphilum litorale* is culturable [83] and next steps should include culturing the strains identified here with the aim of satisfying the Henle-Koch postulates via disease transmission assays.

The other eight late responding, disease outcome-associated ASVs did not differ in abundance between the healthy and diseased experimental doses, indicating that they are mostly likely secondary opportunists. One of these, ASV 134, belongs to the genus *Vibrio*, which has been repeatedly linked to WBD [24, 48, 49], with *Vibrio charcharia* (= *V. harveyi*) initially proposed as the putative pathogen for WBD [35]. Vibrios are well-known marine opportunistic pathogens [89–91] that have been implicated in a variety of other coral diseases [34], including recent links to the highly destructive stony coral tissue loss disease [92–94]. Selwyn *et al.* [44] identified a *Vibrio* sp. (ASV 8) as one of two likely pathogen candidates for WBD in Panama alongside *C. litorale*. While our *C. litorale* ASV differed from Selwyn’s by a single base, our *Vibrio* ASV 134 differed from their top *Vibrio* ASV8 by 21 out of 417 nucleotides (5% different), indicating that different *Vibrio* species or strains are contributing to WBD outcomes across the Caribbean. Moreover, Selwyn *et al.* [45] found that treating coral fragments with antibiotics prior to disease exposure significantly reduced abundances of native *Vibrio* communities, particularly ASV8, and led to decreased WBD transmission. In general, this suggests that *Vibrio* spp. are likely playing a key role as secondary opportunistic members of the WBD pathobiome [43, 49].

The remaining seven late responding, disease-associated opportunists belong to the family *Colwelliaceae* with five ASVs belonging to the genus *Thalassotalea* (ASV 88, 144, 148, 372, 769) and two ASVs being unclassified at the genus level (ASV 166 and ASV 939). *Colwelliaceae* spp. are not well-known marine pathogens, although members of this family have been associated with disease in a variety of marine systems, including lobster shell disease [95] and wasting disease in eelgrass [96]. *Colwelliaceae* has also been associated with several coral diseases, such as WBD [23, 43, 82], stony coral tissue loss disease [93, 97] and white plague-like diseases in the Pacific [98] and the Red Sea [99]. *Colwelliaceae* spp. decompose organic matter [100] and have been shown to respond strongly to the input of dissolved organic matter [101], suggesting that they may be involved in the wasting phase of disease [96]. This role in breaking down organic material may explain the frequent opportunistic associations between *Colwelliaceae* spp. and marine diseases.

## Conclusions

Our 16S rRNA gene amplicon sequencing of staghorn corals with known variation in genotypic disease resistance failed to identify any significant positive or negative associations between a staghorn coral’s native microbiome and its observed resistance to WBD. Instead, we identified nine late responding bacterial ASVs that were strongly associated with WBD outcomes in the tanks, including ASV 65—a *Cysteiniphilum litorale*—as the top pathogen candidate. The identification of *C. litorale* as the putative WBD pathogen in this Florida nursery-based transmission experiment matches recent findings in wild staghorn coral populations in Panama and suggests that highly related *C. litorale* strains likely cause WBD across the Caribbean. To our knowledge, this is the first example of a basin-wide strain-level pathogen association for a coral disease. Efforts should now focus on the cultivation of ASV 65, *C. litorale*, to fulfill the Henle-Koch postulates.

## Supplementary Material

supplemenatry_materials_ycaf247

## Data Availability

The data underlying this article are available as NCBI BioProject PRJNA1279204. Non-genetic data and code for this study are available at https://github.com/VollmerLab/16S_Florida_Tank_Analysis.
